# PART1 and hsa-miR-429-Mediated SHCBP1 Expression Is an Independent Predictor of Poor Prognosis in Glioma Patients

**DOI:** 10.1155/2020/1767056

**Published:** 2020-04-13

**Authors:** Chengmin Xuan, Mingwei Jin, Lei Wang, Shengbai Xue, Qi An, Qingzeng Sun, Lei Wang, Yong Gao

**Affiliations:** ^1^Department of Hematology, Xuzhou Children's Hospital, Xuzhou Medical University, Xuzhou, Jiangsu 221006, China; ^2^Department of Clinical Medicine, Nanjing Medical University, Nanjing, Jiangsu 211166, China; ^3^Department of Orthopaedics, Xuzhou Children's Hospital, Xuzhou Medical University, Xuzhou, Jiangsu 221006, China; ^4^Department of Neurosurgery, Brain Hospital, Affiliated Hospital of Xuzhou Medical University, Xuzhou, Jiangsu 221002, China

## Abstract

Gliomas are the most common primary brain tumors. Because of their high degree of malignancy, patient survival rates are unsatisfactory. Therefore, exploring glioma biomarkers will play a key role in early diagnosis, guiding treatment, and monitoring the prognosis of gliomas. We found two lncRNAs, six miRNAs, and nine mRNAs that were differentially expressed by analyzing genomic data of glioma patients. The diagnostic value of mRNA expression levels in gliomas was determined by receiver operating characteristic (ROC) curve analysis. Among the nine mRNAs, the area under the ROC curve values of only *CEP55* and *SHCBP1* were >0.7, specifically 0.834 and 0.816, respectively. Additionally, *CEP55* and *SHCBP1* were highly expressed in glioma specimens and showed increased expression according to the glioma grade, and outcomes of high expression patients were poor. *CEP55* was enriched in the cell cycle, DNA replication, mismatch repair, and P53 signaling pathway. *SHCBP1* was enriched in the cell cycle, DNA replication, ECM receptor interaction, and P53 signaling pathway. Age, grade, IDH status, chromosome 19/20 cogain, and *SHCBP1* were independent factors for prognosis. Our findings suggest the PART1-hsa-miR-429-SHCBP1 regulatory network plays an important role in gliomas.

## 1. Introduction

Recently, gliomas have received more attention due to the unsatisfactory 5-year survival rates and median survival times of glioma patients [1–5]. However, current comprehensive treatments, comprising surgery, radiotherapy, and chemotherapy, have not achieved the desired therapeutic effects [2, 3]. With the continuous development of genomics, researchers are paying closer attention to the molecular expression changes of glioma patients, such as IDH status, MGMT promoter methylation, and 1p/19q codeletion [6, 7]. Therefore, we explored biomarkers that could guide the early diagnosis, treatment, and prognosis of gliomas through genomic data.

Most of the sequences transcribed from the human genome are long-chain noncoding RNAs (lncRNAs) [8–10]. Increasing evidence has indicated that altered interactions between lncRNAs, miRNAs, and downstream target genes are closely related to tumor development [11–14]. The lncRNAs that regulate miRNA activity by means of “sponge” adsorption are also known as competing endogenous RNAs (ceRNAs) [15]. These lncRNAs act as ceRNAs by competitively binding to miRNAs, thereby regulating protein levels and subsequently cellular behaviors [16, 17]. However, the lncRNAs that act as ceRNAs in tumors are poorly understood.

SHC binding and spindle associated 1 (*SHCBP1*) is located on 16q11.2 and encodes a protein in the Src homolog and collagen homolog (SHC) family that is a target of the SHC adaptor protein [18]. Studies have shown that SHCBP1 plays a role in tumor development that may be related to activation of the FGF [18], NF-*κ*B [19], and/or TGF-*β*1/Smad signaling pathways [20]. Studies have also shown that SHCBP1 is involved in the development of various tumors such as hepatocellular carcinoma [21], glioma [19], breast cancer [22], and gastric cancer [23].

A centrosomal protein of 55 kDa (*CEP55*) is located at 10q23 and is a member of the centrosome-associated protein family [24]. CEP55 is involved in cytokinesis [25]; during late-stage cytokinesis, CEP55 recruits proteins that are directly related to cell membrane separation, such as tumor-susceptibility gene 101 (TSG101) and ALG2-interacting protein X (ALIX) [26, 27]. Regarding tumor development, CEP55 is involved in the development of glioma [28], hepatocellular carcinoma [29], breast cancer [30], lung cancer [31], and ovarian cancer [32]. Although there are reports of a relationship between SHCBP1 and CEP55 in glioma, these have only focused on their roles in the migration and invasion of glioma cells. We used glioma lncRNA and mRNA databases to analyze the role of *SHCBP1* and *CEP55* in glioma and their associations with prognosis.

We started with the lncRNA database to obtain differentially expressed lncRNAs, miRNAs, and mRNAs and to construct lncRNA-miRNA-mRNA regulatory networks. The receiver operating characteristic (ROC) curve showed that *CEP55* and *SHCBP1* had higher predictive values for patient prognosis. Furthermore, *CEP55* and *SHCBP1* were highly expressed in gliomas, showing higher expression in higher grade cases, and high expression patients had poorer prognoses. KEGG enrichment analysis revealed that CEP55 and SHCBP1 are enriched in multiple signaling pathways involved in tumorigenesis and development. Cox analysis found that *SHCBP1* along with age, grade, IDH status, and chromosome 19/20 cogain were independent factors for determining patient prognosis.

## 2. Materials and Methods

### 2.1. Patient Samples

lncRNA and mRNA expression data were obtained from the Gene Expression Omnibus (GEO), Chinese Glioma Genome Atlas (CGGA), and The Cancer Genome Atlas (TCGA) databases. GSE103227 (https://www.ncbi.nlm.nih.gov/geo/query/acc.cgi?acc=GSE103227) contains five nontumor brain tissues and five glioma tissues. GSE103229 (https://www.ncbi.nlm.nih.gov/geo/query/acc.cgi?acc=GSE103229) contains five nontumor brain tissues and five glioma tissues. The GSE4290 and CGGA database information refers to our previous description [33, 34]. TCGA (https://www.cancer.gov/about-nci/organization/ccg/research/structural-genomics/tcga) contains 555 glioma specimens, including 210 in the World Health Organization (WHO) II, 233 in the WHO III, and 112 in the WHO IV. The WHO classification system was used according to our previous description [34]. The Xuzhou Children's Hospital Medical Ethics Committee approved this study protocol.

### 2.2. Identifying Differentially Expressed lncRNAs, miRNAs, mRNAs, and Overlapping Genes

To obtain differentially expressed lncRNAs, miRNAs, and mRNAs, we selected the lncRNA and mRNA databases of GSE103227 and GSE103229 and obtained differentially expressed lncRNAs and mRNAs. GSE103227 was set to logFC ≥ 3, and *p* values < 0.01 were considered statistically significant. GSE103229 was set to logFC ≥ 2, and *p* values < 0.01 were considered statistically significant. Next, we obtained miRNAs that interacted with the differentially expressed lncRNAs using miRcode software (http://www.mircode.org/). We predicted the target genes of these miRNAs using miRDB (http://mirdb.org/), miRTarBase (https://bio.tools/mirtarbase), and TargetScan (http://www.targetscan.org/vert_72/). We only accepted target genes if they were predicted by all three programs. We retained the overlapping mRNAs from these miRNA target genes and the differentially expressed mRNAs from the databases. Then, based on these overlapping mRNAs, we retained lncRNAs and miRNAs to map the ceRNA network using Cytoscape (https://cytoscape.org/). To increase the credibility of these data, we obtained a corresponding lncRNA-miRNA-mRNA regulatory network through the intersections of the respective lncRNAs, miRNAs, and mRNAs of GSE103227 and GSE103229 in R software (https://www.r-project.org/).

### 2.3. ROC Curve Analysis of mRNA

After obtaining differentially expressed mRNAs, we performed ROC curve analysis using R software to obtain a molecular marker that could be used to predict patient prognosis.

### 2.4. Expression Level and Survival Analysis of CEP55 and SHCBP1

To investigate *CEP55* and *SHCBP1* expression levels in nontumor brain tissue and glioma, we used the GSE4290 and CGGA databases to analyze their expression in nontumor brain tissue and different grades of gliomas. Relationships between these variables and patient prognosis were analyzed using GraphPad Prism software (GraphPad Software, Inc., San Diego, CA, USA).

### 2.5. Gene Set Enrichment Analysis (GSEA) and Cox Analysis of CEP55 and SHCBP1

To analyze the signaling pathways regulated by CEP55 and SHCBP1 in glioma, we performed KEGG enrichment analysis by GSEA (http://software.broadinstitute.org/gsea/index.jsp). To further determine whether CEP55 and SHCBP1 can be used as molecular markers to predict patient outcomes, we performed single-factor and multivariate Cox analyses in TCGA glioma database using R software. Multivariate Cox analysis results were presented as forest plots using R software.

### 2.6. Statistical Analysis

GraphPad Prism 6 was used for all statistical analyses. Statistical significance was defined as a two-tailed *p* value < 0.05. Univariate Cox analysis and multivariate Cox analysis were performed using R software, with *p* values < 0.05 considered statistically significant. GSEA was performed using GSEA software, where ∣normalized enrichment score (NES)∣ > 1, NOM *p* value < 0.05, and FDR *q* value < 0.25 were considered to indicate statistical significance.

## 3. Results

### 3.1. Acquiring Differentially Expressed lncRNAs, miRNAs, and mRNAs

**Figure 1:**
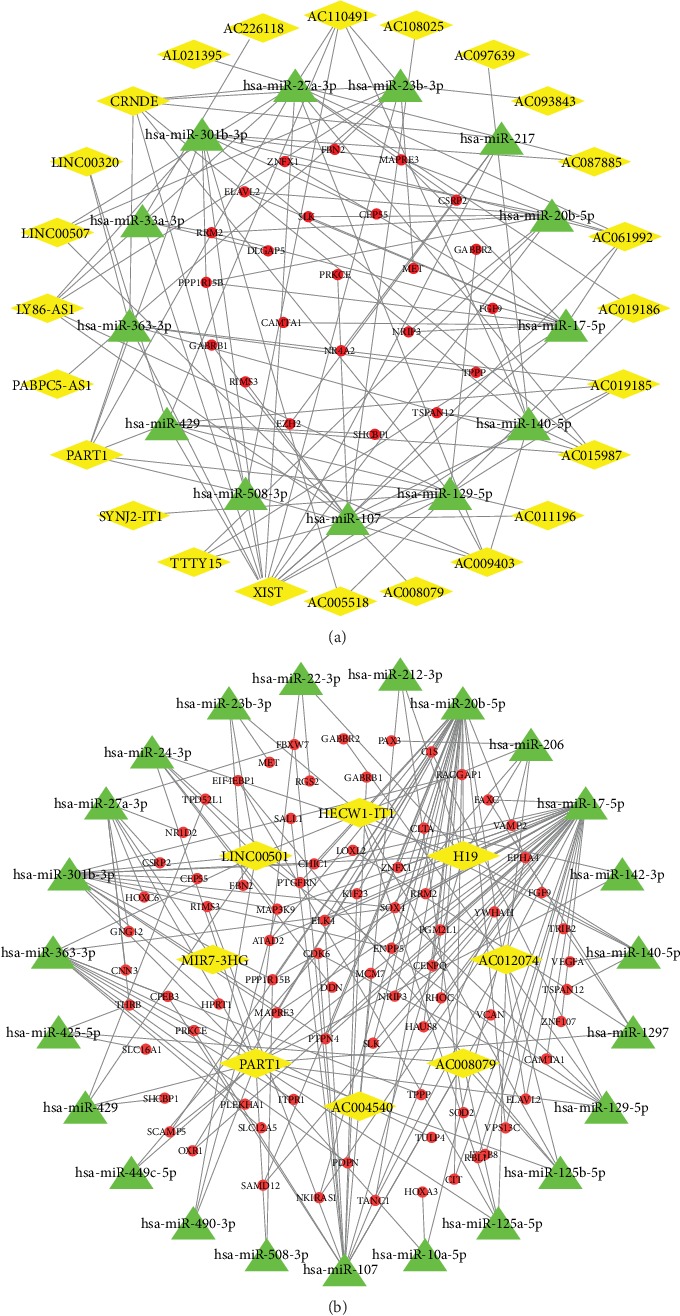
Acquisition of ceRNA networks. (a, b) To obtain lncRNA-miRNA-mRNA interaction networks, we mapped the ceRNA networks in GSE103227 (a) and GSE103229 (b). Yellow represents lncRNAs, green represents miRNAs, and red represents mRNAs.

**Figure 2:**
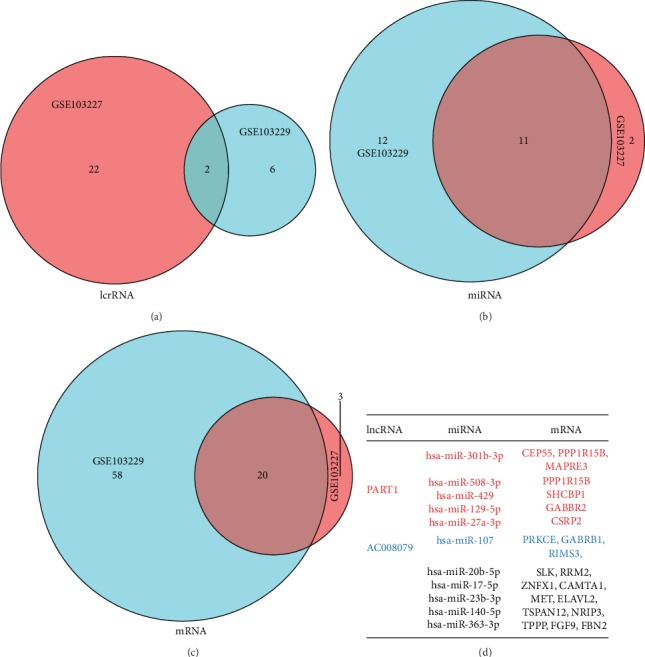
Acquisition of overlapping lncRNAs, miRNAs, and mRNAs. (a–c) To increase data credibility, we cross-validated the differentially expressed lncRNAs (a), miRNAs (b), and mRNAs (c) obtained from GSE103227 and GSE103229 to obtain a more reliable lncRNA-miRNA-mRNA regulatory network. (d) The correspondence between lncRNA-miRNA-mRNA regulatory networks are as follows: red and blue: lncRNA, miRNA, and mRNA have a corresponding relationship; black: miRNA and mRNA have no corresponding relationship.

To obtain lncRNAs, miRNAs, and mRNAs that predict patient prognosis, we analyzed the lncRNA and mRNA expression profiles of human glioblastomas in GSE103227 and GSE103229. In GSE103227, we obtained 24 differentially expressed lncRNAs and 23 differentially expressed mRNAs. Additionally, there were eight and 78 in GSE103229, respectively. These results are shown in the ceRNA network (Figures 1(a) and 1(b)). Based on the differentially expressed lncRNAs, we predicted miRNAs that might interact with them. GSE103227 had 13 such miRNAs, while GSE103228 had 23. To increase the reliability of these data, we obtained the intersection of differentially expressed lncRNAs, miRNAs, and mRNAs from two databases. This revealed two lncRNAs, six miRNAs, and nine mRNAs (Figures 2(a)–2(c)). PART1 interacts with hsa-miR-129-5p, hsa-miR-27a-3p, hsa-miR-301b-3p, hsa-miR-429, and hsa-miR-508-3p. AC008079 interacts with hsa-miR-107. The corresponding mRNAs for these miRNAs are shown in Figure 2(d).

### 3.2. Predicting CEP55 and SHCBP1 Expression, Patient Prognosis, and Participation in KEGG Signaling Pathways

**Figure 3:**
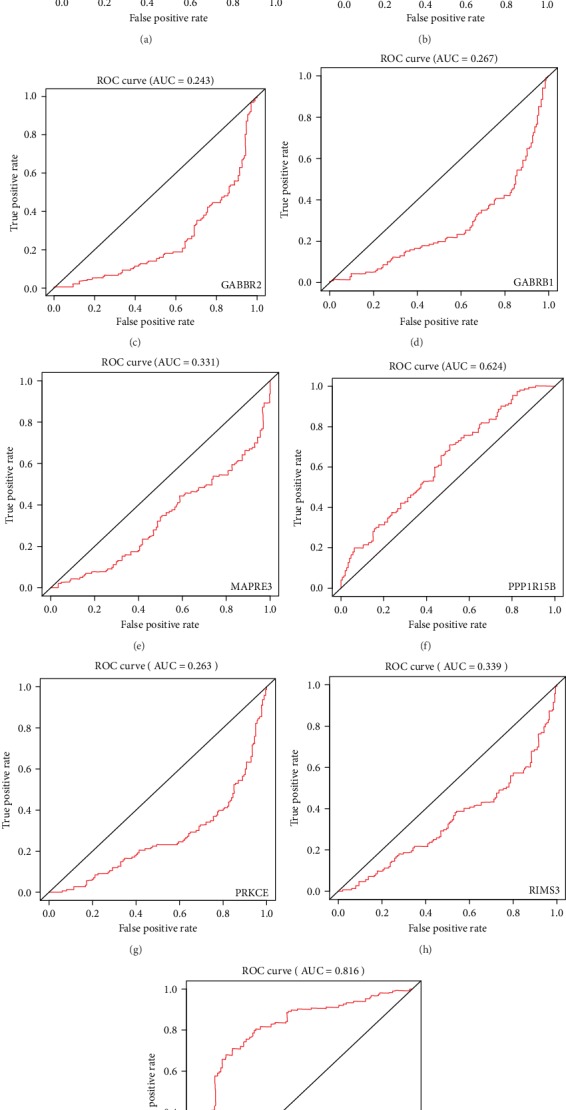
Receiver operating characteristic (ROC) curve analysis of overlapping mRNAs. To investigate the accuracy of overlapping mRNAs as a predictor of patient outcomes, we performed ROC curve analysis of *CEP55* (a), *CSRP2* (b), *GABBR2* (c), *GABRB1* (d), *MAPRE3* (e), *PPP1R15B* (f), *PRKCE* (g), *RIMS3* (h), and *SHCBP1* (i). AUC values represent the area under the curve.

**Figure 4:**
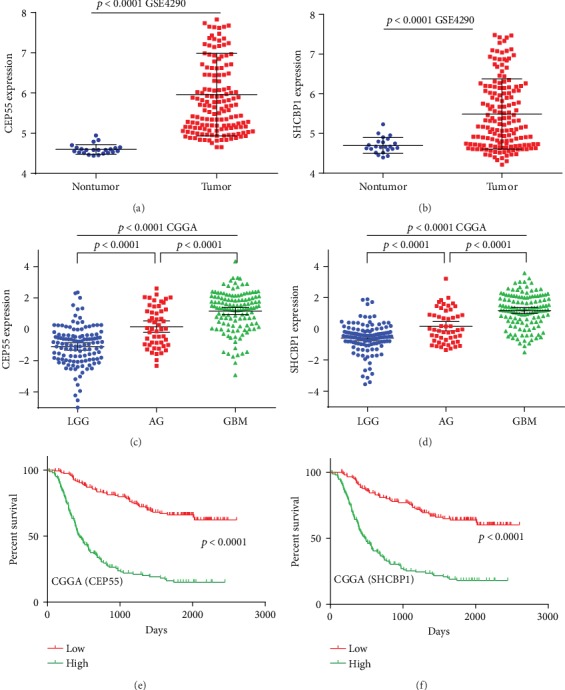
*CEP55* and *SHCBP1* expression levels in gliomas and their relationship to prognosis. (a, b) Expression of *CEP55* (a) and *SHCBP1* (b) in nontumor brain tissue and glioma tissues. (c, d) Expression of *CEP55* (c) and *SHCBP1* (d) in glioma tissues of different levels. (e, f) Kaplan–Meier survival curve analysis of the prognostic significance of *CEP55* and *SHCBP1* expression in glioma patients.

**Figure 5:**
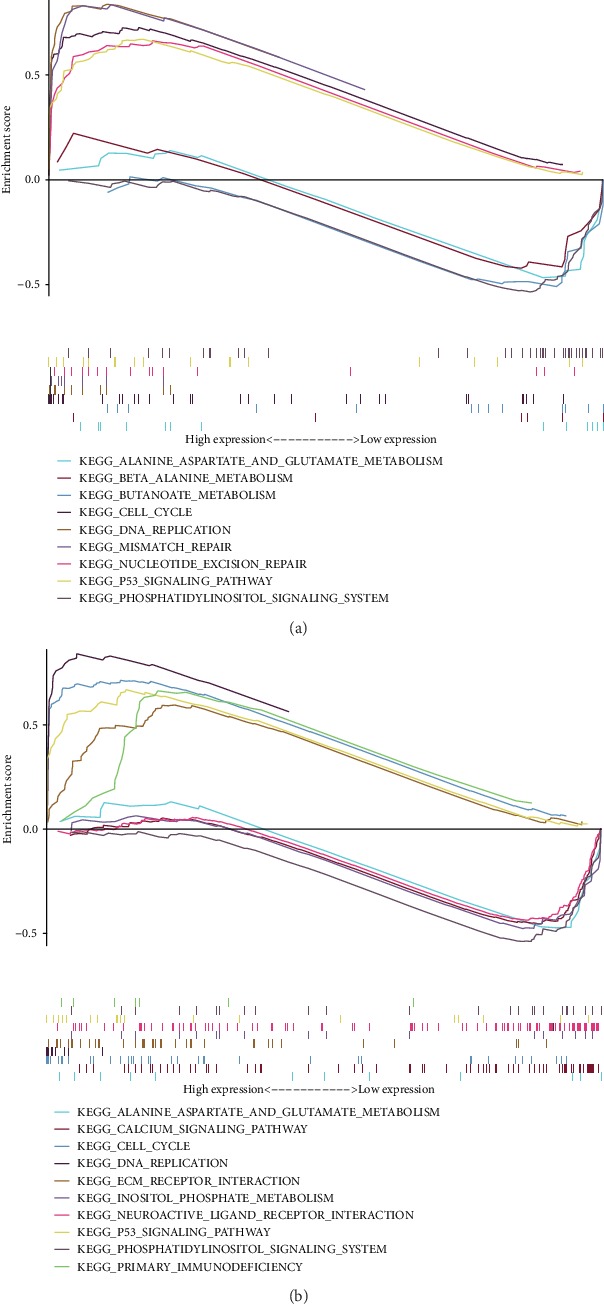
Gene Set Enrichment Analysis (GSEA) of *CEP55* and *SHCBP1*. (a) GSEA found that *CEP55* is enriched in the cell cycle, DNA replication, mismatch repair, and the P53 signaling pathway. (b) GSEA found that SHCBP1 is enriched in the cell cycle, DNA replication, ECM receptor interaction, and P53 signaling pathway.

To investigate the accuracy of differential gene prediction for patient prognosis, we plotted the ROC curves. Among them, *CEP55* and *SHCBP1* had values greater than 0.7. Conversely, *CSRP2*, *GABBR2*, *GABRB1*, *MAPRE3*, *PPP1R15B*, *PRKCE*, and *RIMS3* had values less than 0.7 (Figures 3(a)–3(i)). By ROC curve analysis, we obtained two lncRNA-miRNA-mRNA regulatory networks: PART1-hsa-miR-429-SHCBP1 and PART1-hsa-miR-301b-3p-CEP55. By analyzing the expression of *CEP55* and *SHCBP1* in glioma patients and its correlations to patient prognosis, we found higher expression of *CEP55* and *SHCBP1* in glioma tissues than in nonmalignant tissue (Figures 4(a) and 4(b)) and increased expression with increased glioma grade (Figures 4(c) and 4(d)), and survival analysis found that patients with low *CEP55* and *SHCBP1* expression had better prognoses (Figures 4(e) and 4(f)). To determine the role of CEP55 and SHCBP1 in glioma, we performed KEGG analyses. These revealed that CEP55 is enriched in the cell cycle, DNA replication, mismatch repair, and P53 signaling pathway (Figure 5(a)), while SHCBP1 is enriched in the cell cycle, DNA replication, ECM receptor interaction, and P53 signaling pathway (Figure 5(b)).

### 3.3. Cox Analysis of CEP55 and SHCBP1 for Predicting Patient Prognosis

**Figure 6:**
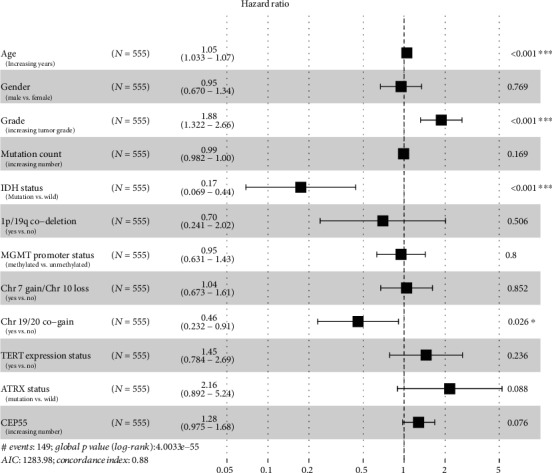
Multivariate Cox analysis of *CEP55*. Forest plots showing multivariate Cox analysis of whether *CEP55* and multiple glioma patient characteristics can be used as independent factors to predict patient prognosis. Yes indicates that the trait is present; no indicates that the trait is absent.

**Figure 7:**
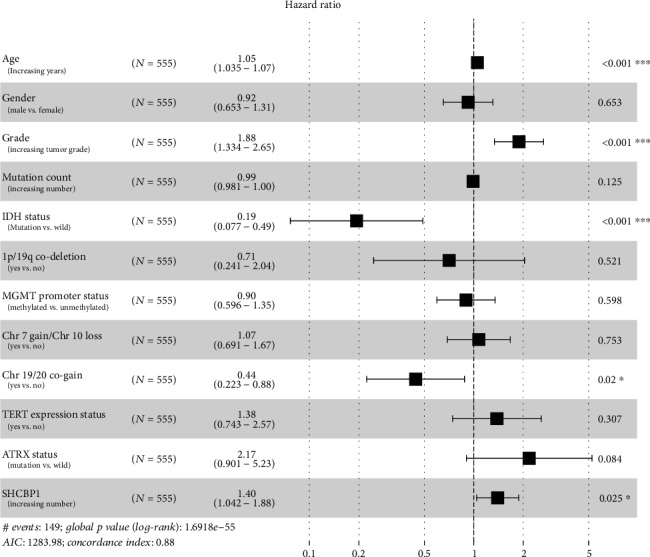
Multivariate Cox analysis of *SHCBP1*. Forest plots showing multivariate Cox analysis of whether *SHCBP1* and multiple glioma patient characteristics can be used as independent factors to predict patient prognosis. Yes indicates that the trait is present; no indicates that the trait is absent.

Through ROC curve analysis, we found that *CEP55* and *SHCBP1* had low false-positive rates as predictors of prognosis. To illustrate the relationship between *CEP55*, *SHCBP1*, and patient prognosis, we performed univariate and multivariate analyses. Univariate Cox analysis showed that except for gender and number of genetic mutations, patient prognosis increased the HR of each additional unit of the corresponding indicator, which was statistically significant. The HR of *CEP55* and *SHCBP1* increased by 2.26 and 2.77, respectively, for each additional unit of expression (Table 1). Multivariate Cox analysis showed that age, grade, IDH status, chromosome 19/20 cogain, and *SHCBP1* were independent predictors of patient outcomes; these results are shown through forest plots (Figures 6 and 7, Table 1). Based on these results and the developed lncRNA-miRNA-mRNA regulatory network, we found that the PART1-hsa-miRNA-429-SHCBP1 signaling pathway plays an important role in glioma development.

## 4. Discussion

Through analyzing the human glioma databases GSE103227 and GSE103229, we obtained differentially expressed lncRNAs and mRNAs. We then found the miRNAs that interacted with these differentially expressed lncRNAs. To increase data, we cross-validated the two database results and obtained two lncRNAs, 11 miRNAs, and 20 mRNAs. After ROC curve analysis, we found that only *CEP55* and *SHCBP1* were prognostic with high accuracy, and the lncRNA-miRNA-mRNA regulatory networks used were PART1-hsa-miR-301b-CEP55 and PART1-hsa-miR-429-SHCBP1. We further analyzed *CEP55* and *SHCBP1* expression in gliomas, their relationships to patient prognosis, and their enriched KEGG signaling pathways. Univariate and multivariate Cox analyses revealed that *SHCBP1*, age, grade, IDH status, and chromosome 19/20 cogain are independent predictors of patient prognosis.

Previous studies have shown that PART1 plays a tumor suppressive function in gliomas [35], but has a procancer effect in bladder cancer [36], non-small-cell lung cancer [37], and colorectal cancer [38]. hsa-miRNA-429 plays an inhibitory role in a number of tumors [39–41], including glioma [42–44]. *SHCBP1* is involved in the development of hepatocellular carcinoma [21], glioma [19], breast cancer [22], and gastric cancer [23]. However, the lncRNAs and miRNAs with which *SHCBP1* interacts and whether it could be used as a prognostic molecular biomarker had been unknown. Based on the bioinformatics analysis of a large number of glioma specimens, we found that PART1 and hsa-miRNA-429 could regulate *SHCBP1* expression and that *SHCBP1* can be used as a molecular biomarker to judge the prognosis of glioma patients. Previous studies have found that *SHCBP1* acts as a cancer-promoting gene in a variety of tumors [19–23], which is consistent with our findings. We also discovered multiple tumor-associated signaling pathways, such as cell cycle and DNA replication, that may lead to glioma development. This is consistent with the results of SHCBP1 activity in hepatocellular carcinoma cells and breast cancer cells, where it regulates cell cycle [21, 22]; SHCBP1 also regulates cell migration via EMT receptor interactions in synovial sarcoma cells [20]. Additionally, we found that *SHCBP1* is low expressed in nontumor brain tissue, but as the level of glioma increases, its expression level is correspondingly increased. Finally, the prognosis of patients with high *SHCBP1* expression is poor, which is consistent with the study by Zhou et al. [19]. In addition to the relationship between *SHCBP1* and patient prognosis, by Cox analysis, we found that *SHCBP1* expression, age, grade, IDH status, and chromosome 19/20 cogain were independent factors associated with patient prognosis. When analyzing IDH (wild) long-term (more than 36 months) and short-term (less than 10 months) survivors of primary glioblastoma, the author found that chromosome 19/20 cogain is a favorable prognostic marker for non-G-CIMP glioma (G-CIMP glioma, CpG island methylator phenotype) patients [45]. Its related mechanism still needs our further research. We obtained results similar to *SHCBP1* for *CEP55*, but it could not be used as an independent factor to judge patient prognosis.

In conclusion, we found that *CEP55* and *SHCBP1* can be used to predict the prognosis of glioma patients with high accuracy and that *SHCBP1* is an independent prognostic factor for glioma patients. *SHCBP1* is highly expressed in glioma patients, later-staged gliomas had higher *SHCBP1* expression, and *SHCBP1* expression levels were negatively correlated with patient survival. SHCBP1 is enriched in multiple signaling pathways such as the cell cycle, DNA replication, ECM receptor interaction, and P53 signaling pathway. The PART1-hsa-miRNA-429-SHCBP1 signaling pathway may play an important role in glioma and provides a new direction for basic research into the underlying biology of gliomas.

## Figures and Tables

**Table 1:** Univariate analysis and multivariate analysis of the correlation of the expression of CEP55 and SHCBP1 with OS among glioma patients.

Parameter	Univariate analysis			Multivariate analysis					
				1			2		
	HR	95% CI	*p*	HR	95% CI	*p*	HR	95% CI	*p*
Age (increasing years)	1.08	1.07-1.10	**2.70** **E** **-32**	1.05	1.03-1.07	**3.37** **E** **-09**	1.05	1.03-1.07	**1.02** **E** **-09**
Gender (male vs. female)	0.95	0.69-1.32	0.795	0.95	0.67-1.34	0.769	0.92	0.65-1.31	0.653
Grade (increasing tumor grade)	5.15	3.92-6.76	**4.33** **E** **-32**	1.86	1.32-2.66	**0.000**	1.88	1.33-2.65	**0.000**
Mutation count (increasing number)	1.00	1.00-1.00	0.340	0.99	0.98-1.00	0.169	0.99	0.98-1.00	0.125
IDH status (mutant ion vs. wild)	0.10	0.07-0.14	**5.85** **E** **-36**	0.17	0.07-0.44	**0.000**	0.19	0.08-0.49	**0.001**
1p/19q codeletion (yes vs. no)	0.24	0.14-0.41	**1.60** **E** **-07**	0.70	0.24-2.02	0.506	0.70	0.24-2.46	0.521
MGMT promoter status (methylated vs. unmethylated)	0.31	0.22-0.43	**2.65** **E** **-12**	0.94	0.63-1.43	0.800	0.90	0.60-1.35	0.598
Chr 7 gain/Chr 10 loss (yes vs. no)	7.97	5.44-11.7	**1.98** **E** **-26**	1.04	0.67-1.61	0.852	1.07	0.69-1.67	0.753
Chr 19/20 cogain (yes vs. no)	2.72	1.46-5.06	**0.002**	0.46	0.23-0.91	**0.026**	0.44	0.22-0.88	**0.020**
TERT expression status (yes vs. no)	2.37	1.69-3.32	**6.30** **E** **-07**	1.45	0.78-2.69	0.236	1.38	0.74-2.57	0.307
ATRX status (mutant ion vs. wild)	0.44	0.30-0.65	**3.16** **E** **-05**	2.16	0.89-5.24	0.088	2.17	0.90-5.23	**0.084**
CEP55 (increasing number)	2.26	1.94-2.64	**8.37** **E** **-26**	1.28	0.97-1.68	0.076			
SHCBP1 (increasing number)	2.77	2.31-3.32	**4.95** **E** **-28**				1.40	1.04-1.88	**0.025**

1 and 2 represent the results of multivariate analysis of CEP55 and SHCBP1, respectively. Bold values indicate *p* < 0.05. HR: hazard ratio; OS: overall survival; CI: confidence interval.

## Data Availability

The authors confirm that all data underlying the findings are fully available without restriction. All relevant data are within the paper.
